# Molecular characterization of multidrug resistant *Enterobacterales* strains isolated from liver and kidney transplant recipients in Spain

**DOI:** 10.1038/s41598-021-90382-5

**Published:** 2021-06-04

**Authors:** Marta Fernández-Martínez, Claudia González-Rico, Mónica Gozalo-Margüello, Francesc Marco, Irene Gracia-Ahufinger, Maitane Aranzamendi, Ana M. Sánchez-Díaz, Teresa Vicente-Rangel, Fernando Chaves, Jorge Calvo Montes, Luis Martínez-Martínez, Maria Carmen Fariñas, ENTHERE Study Group, the Group for Study of Infection in Transplantation of the Spanish Society of Infectious Diseases and Clinical Microbiology (GESITRA-SEIMC) and the Spanish Network for Research in Infectious Diseases (REIPI), Carlos Salas, Carlos Armiñanzas, Francisco Arnaiz de las Revillas, Fernando Casafont-Morencos, Antonio Cuadrado Lavín, Emilio Fábrega, Concepción Fariñas-Álvarez, Virginia Flor Morales, Emilio Rodrigo, Juan Carlos Ruiz San Millán, Marta Bodro, Asunción Moreno, Laura Linares, Miquel Navasa, Frederic Cofan, Fernando Rodríguez, Julián Torre-Cisneros, Aurora Páez Vega, José Miguel Montejo, María José Blanco, Javier Nieto Arana, Jesús Fortún, Rosa Escudero Sánchez, Pilar Martin Dávila, Patricia Ruiz Garbajosa, Adolfo Martínez, Javier Graus, Ana Fernández, Patricia Muñoz, Maricela Valerio, Marina Machado, María Olmedo, Caroline Agnelli Bento, Cristina Rincón Sanz, María Luisa Rodríguez Ferrero, Luis Alberto Sánchez Cámara, José María Aguado, Elena Resino

**Affiliations:** 1 Servicio de Microbiología, Hospital Universitario Marqués de Valdecilla, Santander, Spain; 2 Instituto de Investigación Valdecilla-IDIVAL, Santander, Spain; 3 Servicio de Enfermedades Infecciosas, Hospital Universitario Marqués de Valdecilla, Santander, Spain; 4 Servicio de Microbiología, Centro Diagnóstico Biomédico, Hospital Clínic. ISGlobal, Universidad de Barcelona, Barcelona, Spain; 5 Unidad de Microbiología, Hospital Universitario Reina Sofía, Córdoba, Spain; 6 Instituto Maimónides de Investigación Biomédica de Córdoba (IMIBIC), Córdoba, Spain; 7 Servicio de Microbiología, Hospital Universitario de Cruces, Baracaldo, Vizcaya Spain; 8 Instituto de Investigación Sanitaria Biocruces, Vizcaya, Spain; 9 Servicio de Microbiología, Hospital Universitario Ramón y Cajal, Madrid, Spain; 10 Servicio de Microbiología Clínica y Enfermedades Infecciosas, Hospital General Universitario Gregorio Marañón, Madrid, Spain; 11 Servicio de Microbiología, Hospital Universitario 12 de Octubre, Madrid, Spain; 12 Departamento of Microbiología, Unidad de Microbiología, Hospital Universitario Reina Sofía, IMIBIC, Universidad de Córdoba, Córdoba, Spain; 13 Servicio de Digestivo, Hospital Universitario Marqués de Valdecilla, Santander, Spain; 14 Unidad de Calidad, Hospital Universitario Marqués de Valdecilla, Santander, Spain; 15 Servicio de Nefrología, Hospital Universitario Marqués de Valdecilla, Santander, Spain; 16 Servicio de Enfermedades Infecciosas, Hospital Clínic-IDIBAPS, Barcelona, Spain; 17 Unidad de Hígado, Hospital Clínic-IDIBAPS, Universidad de Barcelona, Barcelona, Spain; 18 Departamento de Nefrología, Hospital Clínic-IDIBAPS, Universidad de Barcelona, Barcelona, Spain; 19 Servicio de Enfermedades Infecciosas, Hospital Universitario Reina Sofía, Córdoba, Spain; 20 Unidad de Enfermedades Infecciosas, Hospital Universitario de Cruces, Barakaldo, Spain; 21 Departamento de Enfermedades Infecciosas, Hospital Universitario Ramón y Cajal, Madrid, Spain; 22 Coordinación de Trasplante, Hospital Universitario Ramón y Cajal, Madrid, Spain; 23 Unidad de Hígado, Hospital Universitario Ramón y Cajal, Madrid, Spain; 24 Departamento de Nefrología, Hospital Universitario Ramón y Cajal, Madrid, Spain; 25 Departamento de Nefrología, Hospital General Universitario Gregorio Marañon, Madrid, Spain; 26 Unidad de Enfermedades Infecciosas, Hospital Universitario, 12 de Octubre, Madrid, Spain

**Keywords:** Microbiology, Antimicrobials, Clinical microbiology

## Abstract

The objective of this study was to analyse the mechanisms of resistance to carbapenems and other extended-spectrum-β-lactams and to determine the genetic relatedness of multidrug-resistant *Enterobacterales* (MDR-E) causing colonization or infection in solid-organ transplantation (SOT) recipients. Prospective cohort study in kidney (n = 142), liver (n = 98) or kidney/pancreas (n = 7) transplant recipients between 2014 and 2018 in seven Spanish hospitals. We included 531 MDR-E isolates from rectal swabs obtained before transplantation and weekly for 4–6 weeks after the procedure and 10 MDR-E from clinical samples related to an infection. Overall, 46.2% *Escherichia coli*, 35.3% *Klebsiella pneumoniae*, 6.5% *Enterobacter cloacae*, 6.3% *Citrobacter freundii* and 5.7% other species were isolated. The number of patients with MDR-E colonization post-transplantation (176; 71.3%) was 2.5-fold the number of patients colonized pre-transplantation (71; 28.7%). Extended-spectrum β-lactamases (ESBLs) and carbapenemases were detected in 78.0% and 21.1% of MDR-E isolates respectively. In nine of the 247 (3.6%) transplant patients, the microorganism causing an infection was the same strain previously cultured from surveillance rectal swabs. In our study we have observed a low rate of MDR-E infection in colonized patients 4–6 weeks post-transplantation. *E. coli* producing *bla*_CTX-M-G1_ and *K. pneumoniae* harbouring *bla*_OXA-48_ alone or with *bla*_CTX-M-G1_ were the most prevalent MDR-E colonization strains in SOT recipients.

## Introduction

The prevalence of multidrug-resistant *Enterobacterales* (MDR-E) as a cause of infection in solid-organ transplant (SOT) patients is progressively increasing, mainly during the first month post-procedure. These infections represent an important cause of morbidity and mortality^1,2^. Studies examining the outcome of infection caused by extended-spectrum β-lactamase (ESBL)-producing *Enterobacterales* among SOT recipients report mortality rates ranging from 5 to 20%^3^. Carbapenemase-producing *Enterobacterales* infections are associated with high mortality among SOT recipients^4^.

Most MDR-E are organisms producing ESBLs, carbapenemases or AmpC enzymes (by either plasmid-borne genes or derepressed or hyperexpressed chromosomal genes). Among the numerous ESBLs, CTX-M, TEM and SHV are the most commonly detected^5^. OXA-48-like enzymes along with VIM and KPC are the three most common carbapenemase genes identified in Spain^6^.

Pre-transplant colonization by MDR-E has been described as an important risk factor for infection after SOT^7^. Several studies recommend the screening of patients at high risk for MDR-E colonization, including SOT recipients^8,9^. According to these data, it seems advisable to obtain rectal swabs from SOT recipients at the time of transplantation to assess intestinal colonization by MDR-E. Subsequently, surveillance cultures may be recommended depending on the local epidemiological pattern and individual risk factors^1^.

The objective of this study was to characterize the mechanisms of resistance to extended-spectrum cephalosporins and carbapenems and to determine the genetic relatedness of MDR-E involved in both colonization and infection in SOT recipients.

## Results

### Study population and bacterial strains

A total of 541 MDR-E isolates were obtained from 247 patients with kidney (n = 142), liver (n = 98) or both kidney and pancreatic transplants (n = 7). Of these, 531 were isolated from rectal samples (460 isolates were obtained post-transplantation; 71, pre-transplantation) and 10 were isolated from clinical samples (Table 1).

**Table 1:** Distribution of 541 multidrug-resistant *Enterobacterales* recovered from rectal swabs according to the pre and post-transplantation period and from clinical samples.

	Rectal swabs (n = 531)		Infection (n = 10)	*P*-value
	Pre-transplant (n = 71)	Post-transplant (n = 460)		
*E. coli* (250)	46 (64.8%)	202 (43.9%)	2 (20.0%)	0.059
*K. pneumoniae* (191)	12 (16.9%)*	174 (37.8%)*	5 (50.0%)	**0.011**
*E. cloacae* (35)	5 (7.0%)	28 (6,1%)	2 (20.0%)	0.771
*C. freundii* (34)	5 (7.0%)	29 (6.3%)	0	0.825
Other (31)	3 (4.3%)	27 (5.9%)	1 (10.0%)	0.595

*Statistically significant differences (bold type) were found between pre- and post-transplant rectal swabs.

Other species included: 9 *Morganella morganii*, 7 *Klebsiella oxytoca*, 6 *Proteus mirabilis*, 5 *Enterobacter aerogenes*, 2 *Citrobacter braakii*, 1 *Enterobacter asburiae* and 1 *Citrobacter koseri.*

The microorganisms recovered were the following: *E. coli* (46.2%, n = 250), *K. pneumoniae.*

(35.3%, n = 191), *E. cloacae* (6.5% n = 35), *C. freundii* (6.3%, n = 34) and other species (5.7%, n = 31, corresponding to 9 *Morganella morganii*, 7 *Klebsiella oxytoca*, 6 *Proteus mirabilis*, 5 *Enterobacter aerogenes*, 2 *Citrobacter braakii*, 1 *Enterobacter asburiae* and 1 *Citrobacter koseri*).

Table 2 shows the distribution of 541 MDR-E isolates recovered from patients according to transplant type. Overall, the isolation rates of *E. coli* and *E. cloacae* in kidney recipients were significantly higher than those of liver recipients (143/250, 57.2% vs 104/250, 41.6%, *P* = 0.042 and 22/35, 62.9% vs 9/35, 25.7%, *P* = 0.050 respectively). *K. pneumoniae* was significantly more prevalent in rectal swabs collected post-transplantation (174/460, 37.8%) compared with pre-transplantation (12/71, 16.9%) (*P* = 0.011). No significant differences were found in the prevalence of the other bacterial species between pre- or post-transplantation rectal swabs (Table 1).

**Table 2:** Distribution of 541 multidrug-resistant *Enterobacterales* recovered from 247 patients according to transplant type.

	Transplant type			
	Kidney (n = 142)	Liver (n = 98)	Kidney /pancreas (n = 7)	*P*-value
*E. coli* (250)	143 (57.2%)*	104 (41.6%)*	3 (1.2%)	**0.042**
*K. pneumoniae* (191)	83 (43.5%)	99 (51.8%)	9 (4.7%)	0.328
*E. cloacae* (35)	22 (62.9%)*	9 (25.7%)*	4 (11.4%)	**0.050**
*C. freundii* (34)	18 (53.0%)	15 (44.1%)	1 (2.9%)	0.668
Other (31)	16 (51.6%)	15 (48.4%)	0	0.883
Total	282	242	17	

*Statistically significant differences (bold type) were found in the prevalence of bacterial species between kidney and liver transplant.

Other species included: 9 *Morganella morganii*, 7 *Klebsiella oxytoca*, 6 *Proteus mirabilis*, 5 *Enterobacter aerogenes*, 2 *Citrobacter braakii*, 1 *Enterobacter asburiae* and 1 *Citrobacter koseri.*

### Antimicrobial susceptibility testing

The activities of the 24 antibiotics against the species most frequently isolated (i.e., *E. coli*, *K. pneumoniae*, *E. cloacae* and *C. freundii*) among the 345 MDR-E isolates (one isolate per REP-PCR pattern/preliminary antibiogram and patient) are presented in Supplementary Table 1.

Overall, the resistance rates of the selected MDR-E were 100% to amoxicillin, 98.6% to piperacillin and between 48.4% and 96.8% to cephalosporins. MIC_90_ values of penicillins, penicillin-β-lactamase inhibitor combinations, cephalosporins and aztreonam were > 256 mg/L. The following resistance rates to carbapenems were observed: 4.1% to meropenem, 7.2% to imipenem and 25.8% to ertapenem. Percentages of resistance to aminoglycosides were greater than 37.7% with the exception of amikacin (2.9% resistance rate). Colistin showed the lowest MIC_90_ (1 mg/L). *K. pneumoniae* (70.2%) exhibited increased resistance to fosfomycin compared with *E. coli* (9.2%) or other species.

*K. pneumoniae* exhibited increased rates of resistance compared with other organisms, with more than half of the isolates showing resistance to all the antibiotics tested except imipenem (4.1%), amikacin (4.1%), meropenem (8.3%) and colistin (10.7%).

### Genes encoding ESBLs, AmpC and carbapenemases

The distribution of isolates with genes encoding ESBLs, plasmid-mediated AmpC or carbapenemases or hyperproducing AmpC among 345 MDR-E isolates is shown in Table 3. In all, 269 strains (78.0%) harboured ESBLs genes, CTX-M-group-1 being the most prevalent (53.3%) followed by CTX-M-group-9 in 15.4% of isolates. Detailed information on the exact ESBLs identified is presented in Table 4. Carbapenemases were detected in 73 MDR-E isolates (21.1%). Here, *bla*_OXA-48_ was the most frequently identified gene (16.5% of the strains), noted mainly in *K. pneumoniae,* followed by VIM-1 (3.8%) and KPC-2 (0.9%). No other carbapenemase genes were detected. Among ESBL-producers, 2.1% (n = 3) *E. coli*, 47.3% (n = 52) *K. pneumoniae* and 11.1% (n = 1) *E. cloacae* harboured a carbapenemase gene. In 93.0% (53/57) of the isolates producing OXA-48 (51 K*. pneumonia*e and 2 *E. coli*), CTX-M-15 was also detected. One *E. coli* harboured VIM-1 plus CTX-M-14, and one *K. pneumoniae* isolate produced both KPC-2 and CTX-M-15 (Supplementary Information files).

**Table 3:** Distribution of antibiotic resistance genes encoding ESBLs, AmpC and carbapenemases detected in 345 MDR-E isolates from patients with kidney, liver or combined kidney/pancreas transplant.

Species (n)	*bla*_CTX-M_ (238)			TEM (4)	SHV (27)	AmpC-producing (47)		Carbapenemases (73)			Other^a^ (12)
	CTX-M G1 (184)	CTX-M G9 (53)	CTX-M G8 (1)			Plasmid	Chromosomal hyperproduction	OXA-48 (57)	VIM-1 (13)	KPC-2 (3)	
*E. coli* (152)	75 (49.3%)	40 (26.3%)	1 (0.6%)	3 (2.0%)	22 (14.3%)	4 (2.6%)		3 (2.0%)	2 (1.3%)	–	5 (3.3%)
*K. pneumoniae* (121)	101 (83.5%)	4 (3.3%)	–	1 (0.8%)	4 (3.3%)	–		53 (43.8%)	4 (3.3%)	3 (2.5%)	3 (2.5%)
*E. cloacae* (26)	2 (7.7%)	6 (23.1%)	–	–	1 (3.8%)		15 (57.7%)	1 (3.8%)	2 (7.7%)	–	–
*C. freundii* (23)	1 (4.3%)	2 (8.7%)	–	–	–		19 (82.6%)	–	1 (4.3%)	–	–
Others^b^ (23)	5 (21.7%)	1 (4.3%)	–	–	–		9 (39.1%)	–	4 (17.4%)	–	4 (17.4%)

^a^Other mechanisms were: SHV-1 hyperproduction (4 strains), TEM-1 hyperproduction (4 isolates) and OXA-1 production (4 strains).

^b^Other species included: 7 K*. oxytoca*, 6 M*. morganii*, 4 *E. aerogenes*, 2 *P. mirabilis*, 2 *C. braakii*, 1 *E. asburiae* and 1 *C. koseri.*

**Table 4:** ESBL genes identified in MDR-E isolated from patients with kidney, liver or combined kidney/pancreas transplant.

ESBLs detected	*E. coli* (141)	*K. pneumoniae* (110)	*E. cloacae* (9)	*C. freundii* (3)	Other species (6)
SHV-2		2			
SHV-12	22	2	1		
TEM-19	2				
TEM-52	1				
TEM-169		1			
CTXM-1	6				
CTXM-3	1				
CTXM-8	1				
CTXM-9	7	1	6		1
CTXM-14	22	3		1	
CTXM-15	44	97	1		3
CTXM-27	10				
CTXM-28	1				
CTXM-32	12	1	1	1	2
CTXM-55	6				
CTXM-65	1			1	
CTXM-138	4	2			
CTXM-156		1			
CTXM-182	1				

Plasmid-mediated AmpC production was observed in four *E. coli* isolates (2.6%) (3 CIT and one DHA producers), while the hyperproduction of chromosomal-AmpC was detected in *E. cloacae* (57.7%), *C. freundii* (82.6%) and other MDR-E species (39.1%).

Other mechanisms of resistance to ESBLs could be inferred by interpretative reading of antibiograms, and included SHV-1 hyperproduction (3 K*. pneumoniae* and 1 *E. coli*), TEM-1 hyperproduction (2 *E. coli* and 2 K*. oxytoca*), and OXA-1 production (2 *E. coli* and 2 K*. oxytoca*).

### Molecular epidemiology

PFGE was performed in 287 isolates: *E. coli* (n = 141), *K. pneumoniae* (n = 100), *E. cloacae* (n = 24) and *C. freundii* (n = 22).

*E. coli* isolates recovered from different hospitals and patients exhibit high genetic diversity (Supplementary Fig. 1). A total of 127 isolates were grouped into 113 pulsotypes, 14 of which were non-typeable.

The molecular typing of *K. pneumoniae* (n = 100) revealed 57 different PFGE-patterns, showing a heterogeneous picture (Supplementary Fig. 2). One strain was non-typeable. There were 44 pulsotypes with a single isolate and 13 clusters with two or more isolates. In general, *K. pneumoniae* strains exhibited greater similarity in PFGE-patterns compared with *E. coli* isolates with rates of 1.737 and 1.124, respectively. The predominant clone (#22) included 29 isolates belonging to patients from the same hospital and harboured both CTX-M-15 and OXA-48.

The dendrograms obtained in *E. cloacae* and *C. freundii* demonstrated 21 pulsotypes in both species (Supplementary Figs. 3 and 4), revealing high clonal heterogeneity.

### Association between faecal carriage and infection

The number of patients colonized with MDR-E during the first month post-transplantation (176; 71.3%) was 2.5-fold the number of colonized pre-transplantation patients (71; 28.7%). In both cases, CTX-M-producing *E. coli* was the most prevalent MDR-E isolate.

**Figure 1:**
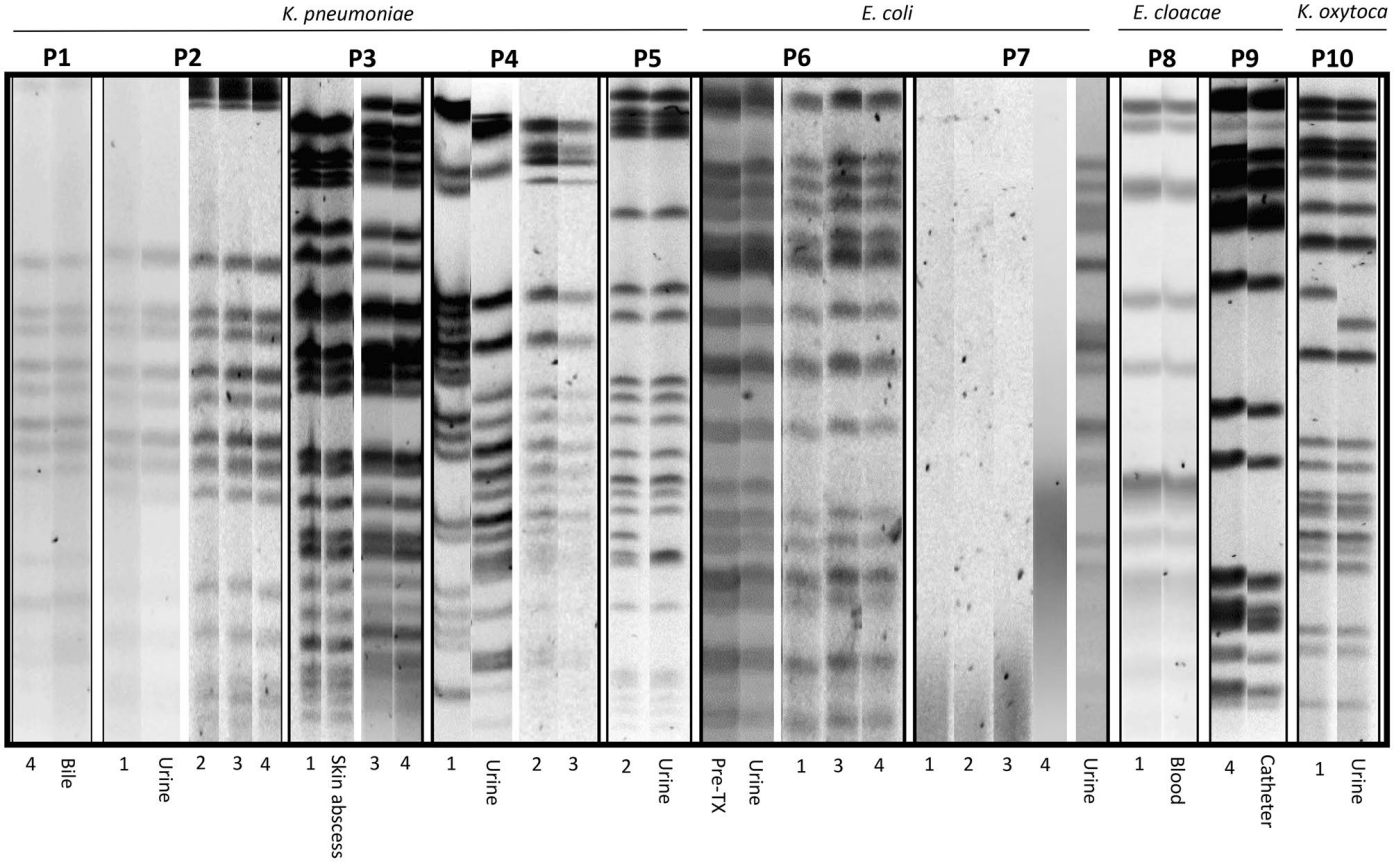
PFGE patterns of MDR-E from rectal swabs obtained pre-transplant (pre-TX) or post-transplant (number indicates weeks for rectal swabs) and from infection-related strains in 10 transplant recipients (P1 to P10 corresponding to Table 5). The PFGE profiles were obtained from different agarose gels and grouped in this figure. (P7: isolates 1, 2, 3 and 4 were non-typeable with *Xba*I).

Ten out of 247 (4.0%) patients colonized by MDR-E developed infections during the post-transplantation follow-up. Isolates from rectal swabs pre- and post-transplant and infections were compared using PFGE and MLST. Table 5 shows the characteristics of these patients and the corresponding organisms. The PFGE patterns are shown in Fig. 1. In 9 of these 10 pairs of MDR-E, the colonization and the infection isolates exhibited identical PFGE-typing and STs.

**Table 5:** Characteristics of the MDR-E isolated by rectal swab and clinical samples from eight kidney and two liver transplant recipients.

Patients	Transplant	Species	Clinical sample	Positive rectal samples (weeks)	Antibiotic resistance profile	Mechanisms of resistance	ST	Hospitals	PFGE-pattern relationship between MDR-E isolated in rectal swab and clinical sample
01	LT	*K. pneumoniae*	Bile	4*	AMX, PIP, AMC, TZP, FOX, CTX, CAZ, FEP, AZT, ETP, IMP, MRP, NAL, CIP, LEV, TO, NET, FOS, TIG	CTX-M-15 + OXA-48	11	H1	Same
02	KT	*K. pneumoniae*	Urine	1*,2,3,4	AMX, PIP, AMC, TZP, FOX, CTX, CAZ, FEP, AZT, ETP, NAL, CIP, LEV, GN, TO, NET, FOS, SXT, TIG. (urine isolate susceptible to GN, TO, NET,)	CTX-M-15 + OXA-48	11	H1	Same
03	KT	*K. pneumoniae*	Skin abscess	1*,3,4	AMX, PIP, AMC, TZP, CTX, CAZ, FEP, AZT, CIP, GN, TO, NET, SXT, TIG	CTX-M-15	429	H2	Same
04	LT	*K. pneumoniae*	Urine	1*,2*,3	AMX, PIP, AMC, TZP, FOX, CTX, CAZ, FEP, AZT, ETP, IMP, MRP, NAL, CIP, LEV, AMK, TO, NET, FOS, TIG, COL	KPC-2	512	H6	Same except RS-1
05	KT	*K. pneumoniae*	Urine	2*	AMX, PIP, AMC, TZP, CTX, CAZ, FEP, AZT, NAL, CIP, LEV, GN, TO, NET, SXT, TIG,	CTX-M-15	307	H6	Same
06	KT	*E. coli*	Urine	Pre-TX*,1,3,4	AMX, PIP, AMC, CTX, CAZ, FEP, AZT, NAL, CIP, LEV, TO NET. (urine isolate susceptible to CAZ and FEP)	CTX-M-15	43	H1	Same
07	KT	*E. coli*	Urine	1*,2,3,4	AMX, PIP, AMC, TZP, CTX, CAZ, FEP, AZT, NAL, CIP, LEV, TO, NET. (strain from RS-1 susceptible to AMC, TO, NET)	CTX-M-15 (urine), CTX-M-32 (RS-1)	621 (urine) and 83 (RS1)	H6	Different
08	KT	*E. cloacae*	Blood	1*	AMX, PIP, AMC, TZP, FOX, CTX, NAL, FOS, TIG	CTX-M-9	97	H1	Same
09	KT	*E. cloacae*	Catheter	4*	AMX, PIP, AMC, FOX, CTX, NAL, FOS	Hyper AmpC	781	H6	Same
10	KT	*K. oxytoca*	Urine	1*	AMX, PIP, AMC, TZP, AZT	Hyper TEM-1	213	H6	Same

*KT* kidney transplant, *LT* liver transplant, *ST* sequence type, *AMC* amoxicillin-clavulanic acid, *AMK* amikacin, *AMX* amoxicillin, *AZT* aztreonam, *CAZ* ceftazidime, *CIP* ciprofloxacin, *COL* colistin, *CTX* cefotaxime, *ERT* ertapenem, *FEP* cefepime, *FOS* fosfomycin, *FOX* cefoxitin, *GN* gentamicin, *IMP* imipenem, *LEV* levofloxacin, *MRP* meropenem, *NAL* nalidixic acid, *NET* netilmicin, *PIP* piperacillin, *RS* rectal sample, *ST* sequence type, *SXT* trimethoprim–sulfamethoxazole, *TIG* tigecycline, *TO* tobramycin, *TZP* piperacillin-tazobactam, *H1* Hospital Universitario Marqués de Valdecilla, *H2* Hospital Clinic, *H6* Hospital Reina Sofia.

*Isolates from the RS that were selected to compare with infection isolate using MLST.

In patient 07, the MDR-E obtained in rectal swabs at 1, 2, 3 and 4 weeks post-TX were non-typeable with *Xba*I. However, the isolate causing urinary infection obtained in the sample presented a clear PFGE pattern, so they were considered not clonally related (Fig. 1). These isolates presented the same ESBL but with different allelic variants (CTX-M-15 and -32).

## Discussion

In this study, the microbiological characteristics of MDR-E isolated from rectal swabs and clinical isolates in SOT patients were analysed. We found high heterogeneity among MDR-E colonizing isolates using a combination of active pre- and post-transplantation surveillance and molecular epidemiology studies.

*E. coli* and *K. pneumoniae* represented the majority of MDR-E colonizing strains in our patients, a finding which is consistent with previous studies performed among SOT recipients^4,10^.

ESBLs were identified in 78.0% of MDR-E, and CTX-M-15 was the most prevalent enzyme. These results are similar to those found in multiple reports worldwide^5,11^. In Spain approximately 20% of infections in SOT recipients are caused by MDR-E, from which 75% are due to ESBL-producing *Enterobacterales*^12^.

Carbapenemases were detected in 21.1% of MDR-E isolates, and OXA-48 was the most common enzyme followed by VIM-1 and KPC-2. Data from the EuSCAPE project in Spain also showed a predominance of OXA-48 and a low prevalence of KPC, VIM or NDM enzymes^13^.

Carbapenems have been traditionally considered the drugs of choice for the treatment of infections caused by ESBL or AmpC-producing enterobacteria; however, their use has contributed to the significant worldwide spread of carbapenem resistance. It is therefore important to consider alternative drugs, such as ceftazidime-avibactam (active against most KPC- and OXA-48-producing *Enterobacterales*), meropenem-vaborbactam and imipenem-relebactam, which have activity against KPC and ESBL-producers^14^. However, MDR-E isolated in our study show a low resistance rate to meropenem (4.1%) and imipenem (7.2%), in contrast to ertapenem (25.8%) for which resistance rates in *K. pneumoniae* and *E. cloacae* were 52.9% and 38.5%, respectively.

Surveillance cultures for detection of colonized patients with MDR-E and implementation of contact precautions, among other measures, have allowed a reduction of the infection rate, both in outbreak and in endemic settings^9,15^.

Post-transplantation complications in SOT patients were associated with both MDR-E colonization and infection, suggesting that in-hospital acquisition of MDR-E in the early post-transplantation period plays an important role^16,17^. In this respect, MDR-E colonization after SOT transplantation has been associated with a subsequent infection at a 1:7 ratio of infected: colonized patients for carbapenem-resistant *Enterobacterales* and 1:4 for organisms resistant to third-generation cephalosporins^17^. In this study, we observed a low rate of infection with MDR-E (4.0%) in colonized patients during follow-up in the first six weeks post-transplantation. In an Italian cohort study, infections caused by KPC-producing *K. pneumoniae* were diagnosed in 29.2% of the liver transplant recipients who were colonized^7^. Other studies have also described higher infection rates (17%-48%) in colonized liver transplant patients^9,16,18^.

Molecular typing of our isolates (PFGE and MLST) revealed significant genetic diversity. Bert et al. also found high clonal diversity between ESBL-producing *Enterobacterales* obtained from colonization and infection after liver transplantation^18^. These findings indicate that MDR-E infections in our patients were not related to the hospital spread of specific clones. The high-risk ST-11 K*. pneumoniae* strain has been frequently detected worldwide as a successful virulent pathogen with determinants for resistance, including VIM, OXA-48^19^ and KPC-production^20^. In our study, all ST11 strains carried CTXM-15 and OXA-48. In recent years, new international drug-resistant lineages have emerged. Among these, *K. pneumoniae* ST307 and KPC-producing *K. pneumoniae* ST512 have been recognized in several countries including Spain^20,21^. ST307 was strongly associated with the diffusion of CTX-M-15, and in France, it has recently been defined as a new successful high-risk clone associated with the dissemination of KPC genes^22^. Both clones were detected as a cause of urinary infection in two patients in our study.

Some study limitations should be considered when extrapolating our results. First, we only collected rectal swabs, which may be inadequate for detecting resistant pathogens present in small amounts. Nevertheless, there is evidence that rectal swabs are more sensitive than other anatomical sites for detecting MDR-E. Secondly, we performed molecular characterization of 541 MDR-E corresponding to 80.2% of colonized patients pre or post-transplantation. Some hospitals did not send all MDR-E isolated from patients enrolled in the study to the coordinating center. And finally, this is a study restricted to a single country, a fact that may limit the extrapolation of its results to other territories. However, it is a prospective study conducted in seven University hospitals in southern Europe where a pioneering transplant program is implemented, and our results may be useful for comparing the evolution of MDR-E among SOT recipients with neighbouring countries.

In summary, molecular typing of MDR-E revealed significant genetic diversity. *E. coli* producing *bla*_CTX-M-G1_ was the principal MDR-E colonizing strain in liver and/or kidney transplant recipients, followed by *K. pneumoniae* harbouring *bla*_OXA-48_ alone or with *bla*_CTX-M-G1._ Rectal colonization by MDR-E increased in the first month after transplantation compared to before transplantation; however, this was not reflected in the infections since there was a low rate of infection by MDR-E during the six-week post-transplantation follow-up.

## Materials and methods

### Study population and setting

This prospective cohort study, which is part of a prospective multicentre cohort study of intestinal colonization by MDR-E in kidney, liver or combined kidney and pancreas transplant recipients in seven Spanish University Hospitals (The ENTHERE study), was conducted between 29 August 2014 and 9 April 2018^23–25^. Adult patients aged 18 years or older undergoing liver, kidney, or pancreas transplantation during the indicated period were included. Patients were followed up 24–48 h before transplantation, weekly until 6 weeks after transplantation, or until death, if this occurred within the indicated period. Intestinal colonization was defined as the isolation of MDR-E in a rectal swab. Infections were defined according to CDC criteria^26^.

Of the 931 patients included in the study, no MDR-E were isolated from any of the rectal swabs collected pre- or post-transplantation in 623 patients. In 308 patients in whom MDR-E was isolated in pre- and post-transplant samples, only 247 patient samples were sent to the coordination center for further analysis.

### Bacterial strains

A total of 531 MDR-E isolates, defined as producing one or more ESBLs, plasmid-mediated or derepressed AmpC or carbapenemases, were included in this study. Rectal swabs were taken 24–48 h pre-transplantation and weekly up to 6 weeks post-transplantation and inoculated onto both ChromID-ESBL and ChromID-CARBA agar plates (BIOMERIEUX, Marcy L’Étoile, France). During the 6 weeks of follow-up, 10 isolates from patients who developed MDR-E infections were also included.

### Antimicrobial susceptibility testing

All 541 MDR-E isolates underwent susceptibility testing for 24 antimicrobials, including amoxicillin, amoxicillin-clavulanic acid (2 mg/L), piperacillin, piperacillin-tazobactam (4 mg/L), cefoxitin, cefotaxime, ceftazidime, cefepime, aztreonam, imipenem, meropenem, ertapenem, amikacin, gentamicin, tobramycin, netilmicin, arbekacin, nalidixic acid, ciprofloxacin, levofloxacin, trimethoprim-sulfamethoxazole, fosfomycin, tigecycline, and colistin, by standardized broth-microdilution methods according to CLSI guidelines^27^. The results were interpreted using EUCAST clinical breakpoints^28^. No breakpoints have been established by EUCAST for arbekacin and nalidixic acid, so the MIC values have been specified. *E. coli* ATCC 25922, *E. coli* ATCC 35218, and *P. aeruginosa* ATCC 27853 were used as quality control strains. Compounds with new β-lactamase inhibitors, such as ceftazidime-avibactam and meropenem-vaborbactam, were not available when the study was planned and consequently could not be tested.

### Molecular fingerprinting

Genetic relatedness was first studied by repetitive-extragenic-palindromic PCR (REP-PCR)^29^ in all 541 MDR-E. Subsequently, the clonal relationship was also determined by pulsed-field gel electrophoresis (PFGE) in at least one strain per patient or strains from the same patient if they had two or more differing bands on REP-PCR.

Bacterial DNA was embedded in agarose plugs and digested with 20 U of *XbaI* at 37ºC overnight. The fragments were separated in 1% agarose gel at 6 V/cm and 14 °C with 0.5xTBE buffer using the CHEF-DRII-system (BIO-RAD, California, USA). Pulse times ranged from 1-5 s for 7 h and 15-35 s for 16 h.

PFGE-patterns were analysed with Fingerprinting II v4.5 software (BIO-RAD). Isolates were classified as indistinguishable if they showed > 95% similarity, as closely related subtypes with 85–95% similarity, and as different strains with similarity < 85%.

### Molecular characterization of resistance genes

In a selection of 345 MDR-E isolates (one isolate per REP-PCR-pattern/patient and antibiogram pattern), standard-PCR was used to detect the presence of genes encoding ESBLs (*bla*_TEM_, *bla*_SHV_ and *bla*_CTX-M_). PCR to detect carbapenemase genes (*bla*_KPC_, *bla*_VIM_, *bla*_IMP_, *bla*_NDM_ and *bla*_OXA-48_) was performed in 90 out 345 MDR-E isolates with a meropenem MIC > 0.125 mg/L, as recommended by EUCAST. In 59 out of 345 isolates not producing an ESBL or carbapenemase gene, multiplex-PCR was performed to detect plasmid-mediated AmpC (*bla*_CIT_, *bla*_FOX_, *bla*_MOX_, *bla*_DHA_, *bla*_ACC_ and *bla*_EBC_).

The primers and PCR conditions used are provided in Supplementary Table 2 online. Representative amplification products were sequenced and analysed in GenBank (www.ncbi.nlm.nih.gov/BLAST).

### Multilocus Sequence Typing (MLST)

MLST was determined for 21 MDR-E isolates collected from 10 patients, including 10 isolates from post-transplantation clinical samples related to an infection and 11 isolates that colonized these infected patients, corresponding to the first rectal swab from which MDR-E were obtained.

Four *Escherichia coli* and 11 *Klebsiella pneumonia*e isolates were assessed using the Pasteur Institute protocols (https://bigsdb.pasteur.fr/index.html). For *K. pneumoniae*, the methodology was used as described^30^. PubMLST (https://pubmlst.org/databases/)^31^ was used to determine the sequence types (ST) of 2 *Klebsiella oxytoca* and 4 *Enterobacter cloacae*.

### Ethics statement

The study was performed in accordance with the Declaration of Helsinki. The protocol was approved by the Institutional Ethics Committee at all participating hospitals [Coordinating laboratory: Hospital Marqués de Valdecilla, (Santander); Hospital Clínic (Barcelona); Hospital Cruces (Bilbao); Hospital Gregorio Marañón (Madrid); Hospital 12 de Octubre (Madrid); Hospital Ramón y Cajal (Madrid) and Hospital Reina Sofía (Córdoba)] according to local standards. Informed consent was obtained from each patient.

## Supplementary Information

Supplementary Information 1.

Supplementary Information 2.

Supplementary Information 3.
