# Supplementary material for: Molecular characterization of multidrug resistant Enterobacterales strains isolated from liver and kidney transplant recipients in Spain
Source: Sci Rep. 2021 Jun 4;11:11875. doi: 10.1038/s41598-021-90382-5 (PMC8178318; doi:10.1038/s41598-021-90382-5)

Molecular characterization of multidrug resistant *Enterobacterales* strains isolated from liver and kidney transplant recipients in Spain

Marta Fernández-Martínez*[1,2], Claudia González-Rico[2,3], Mónica Gozalo-Margüello[1,2], Francesc Marco[4], Irene Gracia-Ahufinger[5,6], Maitane Aranzamendi[7], Ana M Sánchez-Díaz[8], Teresa Vicente-Rangel[9], Fernando Chaves[10], Jorge Calvo Montes[1,2], Luis Martínez-Martínez*[5,6,11] and Maria Carmen Fariñas*[2,3] for the ENTHERE Study Group, for the Group for Study of Infection in Transplantation of the Spanish Society of Infectious Diseases and Clinical Microbiology (GESITRA-SEIMC) and the Spanish Network for Research in Infectious Diseases (REIPI).

1-Servicio de Microbiología, Hospital. Universitario Marqués de Valdecilla, Santander, Spain.
2-Instituto de Investigación Valdecilla (IDIVAL), Santander, Spain
3-Servicio de Enfermedades Infecciosas. Hospital Universitario Marqués de Valdecilla, Santander, Spain.
4-Servicio de Microbiología, Centro Diagnóstico Biomédico, Hospital Clínic. ISGlobal, Universidad de Barcelona, Barcelona, Spain
5-Unidad de Microbiología, Hospital Universitario Reina Sofía, Córdoba, Spain.
6-Instituto Maimónides de Investigación Biomédica de Córdoba (IMIBIC).
7-Servicio de Microbiología, Hospital Universitario de Cruces, Baracaldo, Vizcaya, Spain.and Instituto de Investigación Sanitaria Biocruces.
8-Servicio de Microbiología, Hospital Universitario Ramón y Cajal, Madrid, Spain.
9-Servicio de Microbiología Clínica y Enfermedades Infecciosas, Hospital General Universitario Gregorio Marañón, Madrid, Spain
10-Servicio de Microbiología, Hospital Universitario 12 de Octubre, Madrid, Spain.
11-Departamento of Microbiología, Universidad de Córdoba, Spain.
*Corresponding authors

Marta Fernández Martínez, Claudia González-Rico, Luis Martínez-Martínez and Maria Carmen Fariñas contributed equally to this manuscript

Supplementary Figure 1. Dendrogram showing PFGE patterns obtained in 127 *E. coli* isolated from patients with kidney, liver or combined kidney/pancreas transplant.

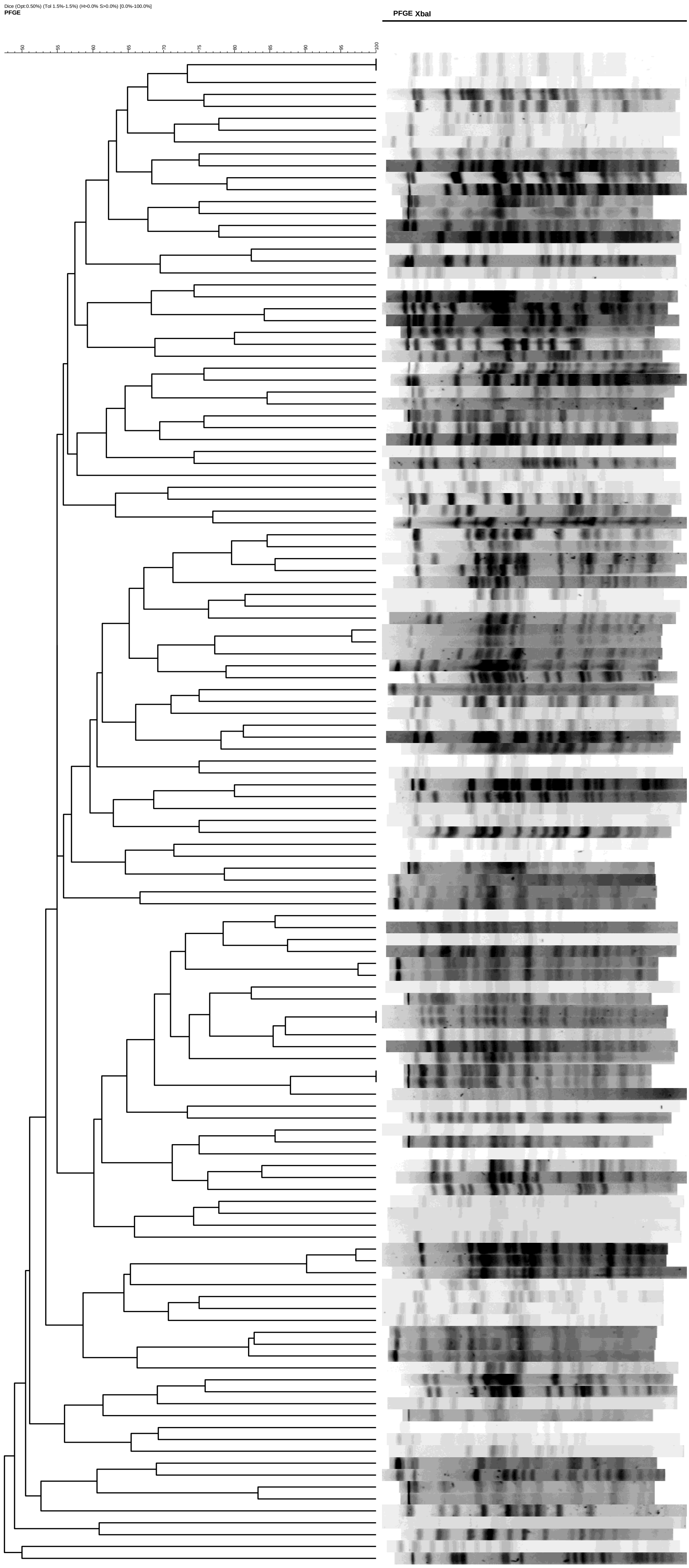

Specie	Hospital	Patient / Visit	Genotype	PFGE
E. coli	HCl	045/5	CTXM-G1	3
E. coli	HCl	049/5	CTXM-G1	3
E. coli	HCl	042/1	SHV	4
E. coli	HURS	023/2	SHV	5
E. coli	HURyC	098/4	CTXM-G1	6
E. coli	HUC	015/3	CTXM-G1	7
E. coli	HUC	043/PreTX	CTXM-G1	8
E. coli	HUMV	041/3	Hiper-AmpCp	9
E. coli	HURyC	085/2	CTXM-G9	10
E. coli	HURS	055/1	Hiper-AmpCp	11
E. coli	HURS	020/4	SHV	12
E. coli	HUMV	165/4	CTXM-G1	13
E. coli	HUMV	118/2	CTXM-G1	14
E. coli	HUMV	121/1	CTXM-G1	15
E. coli	HCl	025/3	CTXM-G1	16
E. coli	HURS	056/PreTX	CTXM-G9	17
E. coli	HUC	002/3	CTXM-G9	18
E. coli	HCl	128/3	Hiper-AmpCp	19
E. coli	HU12O	052/2	CTXM-G1	20
E. coli	HCl	039/2	CTXM-G9	21
E. coli	HURS	064/PreTX	CTXM-G9	22
E. coli	HUC	063/4	CTXM-G1	23
E. coli	HURS	055/2	CTXM-G1	24
E. coli	HUMV	113/4	Others	25
E. coli	HURS	021/2	Others	26
E. coli	HUC	096/PreTX	CTXM-G1	27
E. coli	HUMV	048/PreTX	CTXM-G9	28
E. coli	HUGM	021/4	OXA-48	29
E. coli	HUC	055/PreTX	CTXM-G9	30
E. coli	HUC	111/5	CTXM-G9	31
E. coli	HUMV	137/4	CTXM-G9	32
E. coli	HURS	023/3	CTXM-G1	33
E. coli	HURS	071/2	CTXM-G1	34
E. coli	HUMV	063/PreTX	CTXM-G9	35
E. coli	HCl	136/PreTX	CTXM-G1	36
E. coli	HUMV	100/PreTX	SHV	37
E. coli	HUC	006/4	SHV	38
E. coli	HURS	010/4	SHV	39
E. coli	HUMV	055/PreTX	Hiper-AmpCp	40
E. coli	HUMV	168/PreTX	SHV	41
E. coli	HURS	020/1	Others	42
E. coli	HUMV	007/4	Others	43
E. coli	HUGM	074/2	CTXM-G1	44a
E. coli	HCl	030/PreTX	CTXM-G1	44b
E. coli	HUMV	170/4	CTXM-G1	45
E. coli	HUMV	008/1	CTXM-G9	46
E. coli	HUC	048/PreTX	CTXM-G9	47
E. coli	HCl	127/2	SHV	48
E. coli	HUC	095/PreTX	CTXM-G1	49
E. coli	HUC	098/1	CTXM-G1	49
E. coli	HUC	092/1	CTXM-G9	50
E. coli	HCl	054/3	CTXM-G1	51
E. coli	HCl	054/4	CTXM-G1	52
E. coli	HCl	118/PreTX	SHV	53
E. coli	HURyC	041/4	CTXM-G1	54
E. coli	HU12O	027/1	CTXM-G1	55
E. coli	HURyC	096/3	SHV	56
E. coli	HURS	045/2	CTXM-G1	57
E. coli	HUMV	012/2	CTXM-G1	58
E. coli	HCl	002/PreTX	CTXM-G1	59
E. coli	HURyC	019/1	CTXM-G8	60
E. coli	HUMV	178/1	CTXM-G1	61
E. coli	HUMV	199/PreTX	CTXM-G1	62
E. coli	HURyC	031/PreTX	CTXM-G1	63
E. coli	HUC	030/3	SHV	64
E. coli	HUMV	063/3	SHV	65
E. coli	HCl	029/PreTX	SHV	66
E. coli	HUGM	002/4	CTXM-G1	67
E. coli	HUMV	105/3	CTXM-G1	68
E. coli	HCl	111/4	CTXM-G1	69
E. coli	HCl	082/1	CTXM-G9	70
E. coli	HCl	104/PreTX	CTXM-G1	71
E. coli	HCl	014/PreTX	CTXM-G9	72a
E. coli	HURS	065/2	Hiper-AmpCp	72b
E. coli	HUC	036/4	CTXM-G9	73a
E. coli	HURS	069/PreTX	CTXM-G9	73b
E. coli	HCl	058/2	CTXM-G9	74
E. coli	HCl	060/3	CTXM-G9	74
E. coli	HUC	025/2	CTXM-G1	75
E. coli	HUMV	150/PreTX	CTXM-G1	76
E. coli	HUC	062/3	CTXM-G1	77a
E. coli	HUC	076/PreTX	CTXM-G1	77a
E. coli	HURyC	112/PreTX	CTXM-G1	77b
E. coli	HURS	068/PreTX	CTXM-G1	77c
E. coli	HUC	041/PreTX	CTXM-G9	78
E. coli	HUMV	146/PreTX	CTXM-G1	79a
E. coli	HUMV	146/ Urine	CTXM-G1	79a
E. coli	HUC	087/PreTX	CTXM-G1	79b
E. coli	HURS	003/Urine	CTXM-G1	80
E. coli	HUMV	096/1	SHV	81
E. coli	HUMV	067/PreTX	CTXM-G1	82a
E. coli	HUMV	119/3	CTXM-G1	82b
E. coli	HCl	035/2	CTXM-G1	83
E. coli	HURyC	090/PreTX	CTXM-G1	84
E. coli	HUMV	185/PreTX	CTXM-G1	85
E. coli	HCl	051/2	CTXM-G1	86
E. coli	HURyC	002/1	TEM	87
E. coli	HU12O	026/1	CTXM-G1	88
E. coli	HU12O	022/2	CTXM-G9	89
E. coli	HURyC	021/2	CTXM-G1	90
E. coli	HUC	061/PreTX	SHV	91a
E. coli	HUC	064/3	CTXM-G9	91a
E. coli	HUC	111/PreTX	SHV	91b
E. coli	HUMV	079/PreTX	CTXM-G1	92
E. coli	HUMV	036/3	SHV	93
E. coli	HUMV	038/4	CTXM-G1	94
E. coli	HUMV	061/5	CTXM-G9	95
E. coli	HCl	084/PreTX	CTXM-G1	96
E. coli	HCl	090/4	CTXM-G9	97
E. coli	HCl	112/PreTX	CTXM-G9	98
E. coli	HURyC	068/1	SHV	99
E. coli	HUMV	038/1	SHV	100
E. coli	HCl	044/1	CTXM-G9	101
E. coli	HU12O	044/2	CTXM-G9	102
E. coli	HUMV	139/PreTX	CTXM-G1	103
E. coli	HCl	022/4	SHV	104
E. coli	HUC	050/PreTX	CTXM-G9	105
E. coli	HURyC	011/4	CTXM-G1	106
E. coli	HCl	079/PreTX	CTXM-G1	107
E. coli	HUC	130/PreTX	VIM	108
E. coli	HUMV	108/1	CTXM-G9	109
E. coli	HUMV	108/6	CTXM-G9	110
E. coli	HUGM	036/2	CTXM-G1/OXA-.	111
E. coli	HUGM	007/PreTX	CTXM-G1	112
E. coli	HUGM	008/1	CTXM-G1	113
E. coli	HUC	039/PreTX	CTXM-G9	1
E. coli	HUMV	174/2	CTXM-G1	2

Supplementary Figure 2. Dendrogram showing PFGE patterns obtained in 99 *K. pneumoniae* isolated from patients with kidney, liver or combined kidney/pancreas transplant.

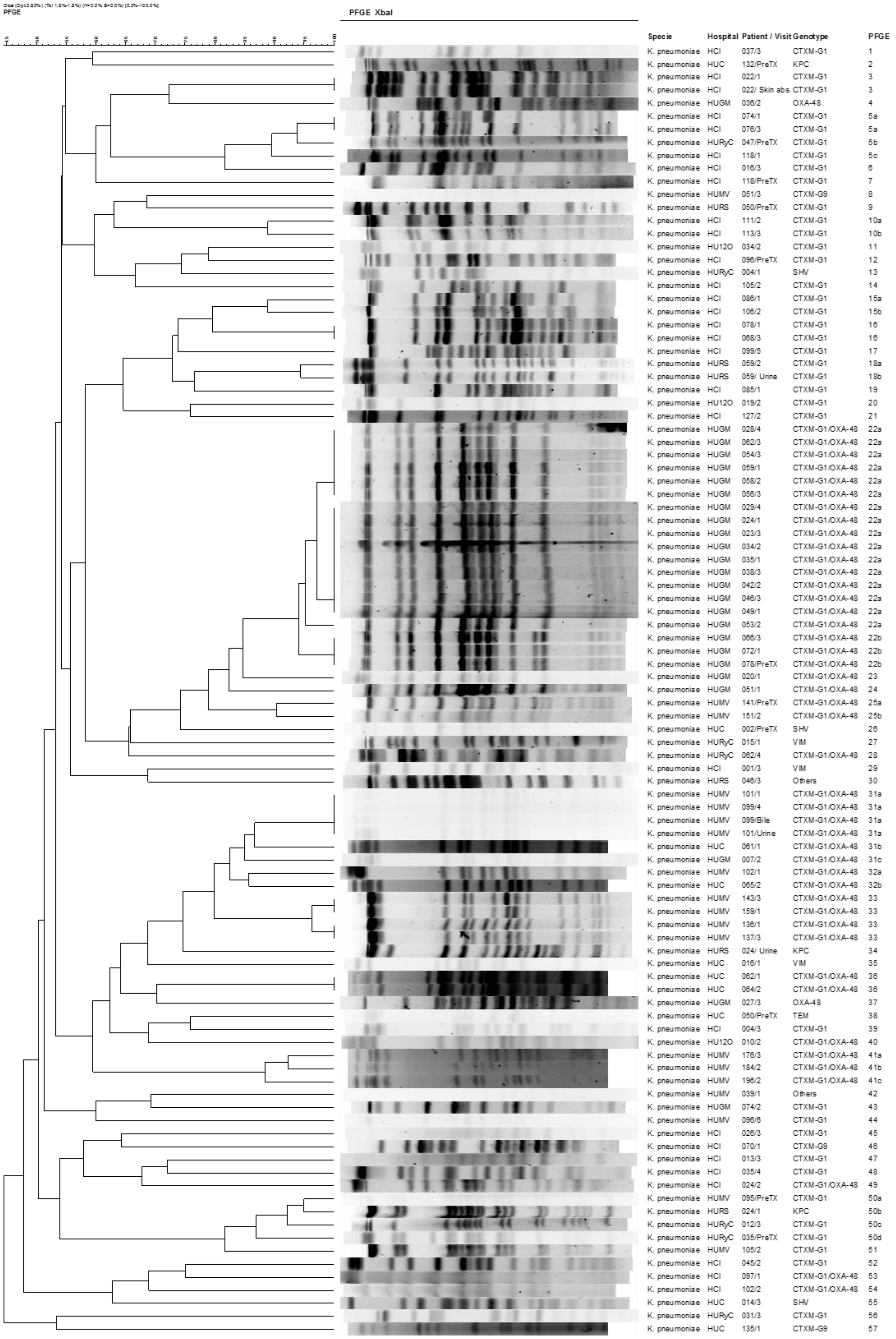

Supplementary Figure 3. Dendrogram showing PFGE patterns obtained in 24 *E. cloacae* isolated from patients with kidney, liver or combined kidney/pancreas transplant.

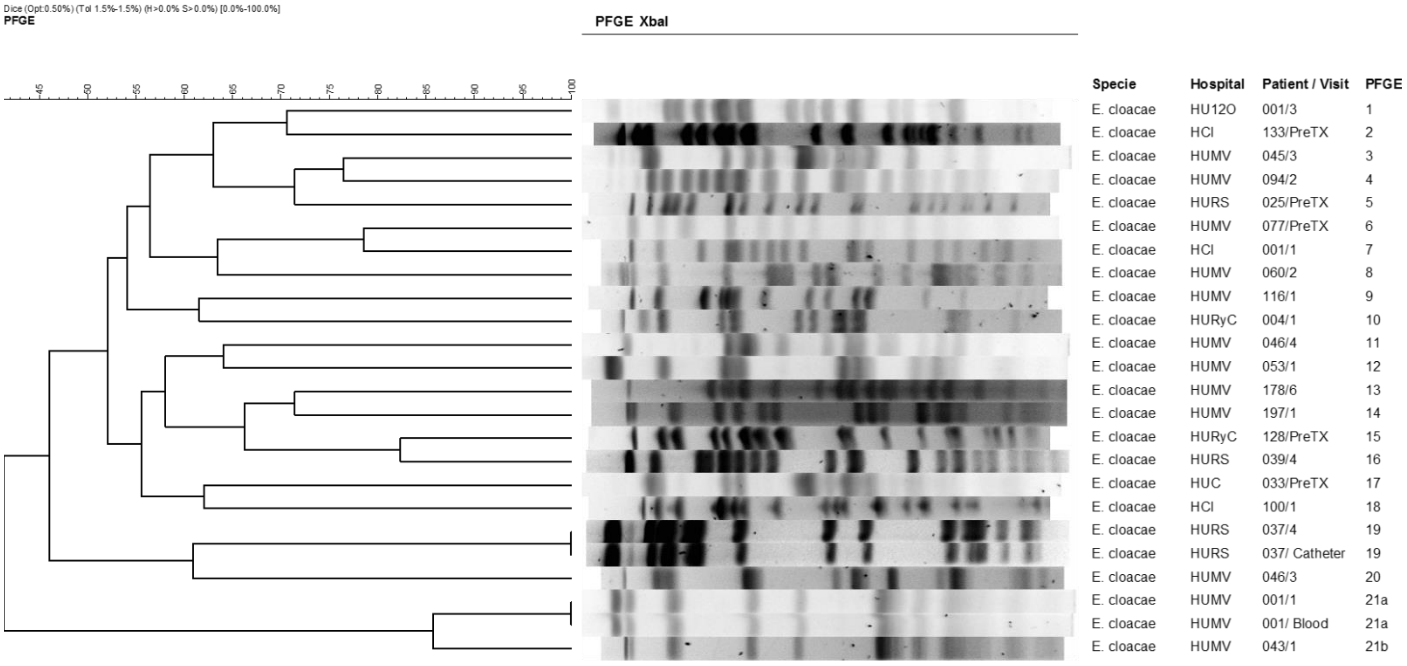

Supplementary Figure 4. Dendrogram showing PFGE patterns obtained in 22 *C. freundii* isolated from patients with kidney, liver or combined kidney/pancreas transplant.

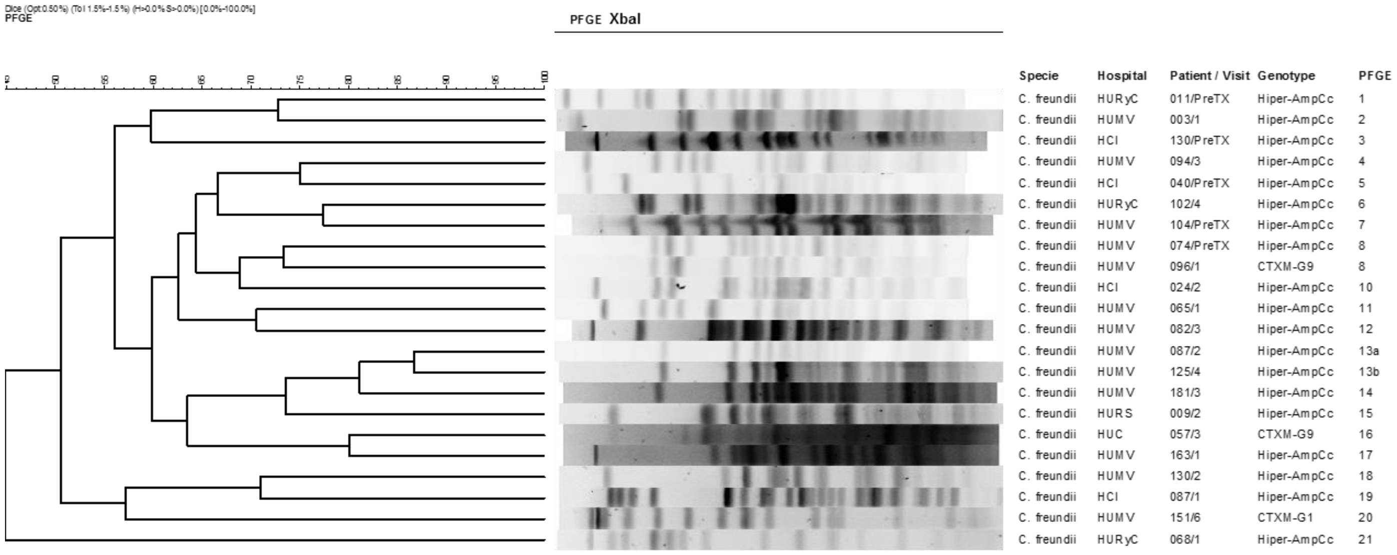

Supplement: Supplementary file 2 — Supplementary Information 2. [file 41598_2021_90382_MOESM2_ESM.pdf]
