# Supplementary material for: Molecular characterization of multidrug resistant Enterobacterales strains isolated from liver and kidney transplant recipients in Spain
Source: Sci Rep. 2021 Jun 4;11:11875. doi: 10.1038/s41598-021-90382-5 (PMC8178318; doi:10.1038/s41598-021-90382-5)
Supplement: Supplementary file 3 — Supplementary Information 3. [file 41598_2021_90382_MOESM3_ESM.pdf]

Molecular characterization of multidrug resistant *Enterobacterales* strains isolated from liver and kidney transplant recipients in Spain

Marta Fernández-Martínez*[1,2], Claudia González-Rico[2,3], Mónica Gozalo-Margüello[1,2], Francesc Marco[4], Irene Gracia-Ahufinger[5,6], Maitane Aranzamendi[7], Ana M Sánchez-Díaz[8], Teresa Vicente-Rangel[9], Fernando Chaves[10], Jorge Calvo Montes[1,2], Luis Martínez-Martínez*[5,6,11] and Maria Carmen Fariñas*[2,3] for the ENTHERE Study Group, for the Group for Study of Infection in Transplantation of the Spanish Society of Infectious Diseases and Clinical Microbiology (GESITRA-SEIMC) and the Spanish Network for Research in Infectious Diseases (REIPI).

1-Servicio de Microbiología, Hospital. Universitario Marqués de Valdecilla, Santander, Spain.
2-Instituto de Investigación Valdecilla (IDIVAL), Santander, Spain
3-Servicio de Enfermedades Infecciosas. Hospital Universitario Marqués de Valdecilla, Santander, Spain.
4-Servicio de Microbiología, Centro Diagnóstico Biomédico, Hospital Clínic. ISGlobal, Universidad de Barcelona, Barcelona, Spain
5-Unidad de Microbiología, Hospital Universitario Reina Sofía, Córdoba, Spain.
6-Instituto Maimónides de Investigación Biomédica de Córdoba (IMIBIC).
7-Servicio de Microbiología, Hospital Universitario de Cruces, Baracaldo, Vizcaya, Spain.and Instituto de Investigación Sanitaria Biocruces.
8-Servicio de Microbiología, Hospital Universitario Ramón y Cajal, Madrid, Spain.
9-Servicio de Microbiología Clínica y Enfermedades Infecciosas, Hospital General Universitario Gregorio Marañón, Madrid, Spain
10-Servicio de Microbiología, Hospital Universitario 12 de Octubre, Madrid, Spain.
11-Departamento of Microbiología, Universidad de Córdoba, Spain.
*Corresponding authors
Marta Fernández Martínez, Claudia González-Rico, Luis Martínez-Martínez and Maria Carmen Fariñas contributed equally to this manuscript

Supplementary Table 1. *In vitro* activity to 24 antimicrobials agents in 345 MDR-E isolated from kidney, liver or combined kidney/pancreas transplant recipients.

	**All isolates (n=345)**				***E. coli*** **(n=152)**				***K. pneumoniae*** **(n=121)**				***E. cloacae*** **(n=26)**				***C. freundii*** **(n=23)**				**Other MDR-E (n=23)**			
Antimicrobial agent	Resistant	MIC_{50} (mg/L)	MIC_{90} (mg/L)	Range (mg/L)	Resistant	MIC_{50} (mg/L)	MIC_{90} (mg/L)	Range (mg/L)	Resistant	MIC_{50} (mg/L)	MIC_{90} (mg/L)	Range (mg/L)	Resistant	MIC_{50} (mg/L)	MIC_{90} (mg/L)	Range (mg/L)	Resistant	MIC_{50} (mg/L)	MIC_{90} (mg/L)	Range (mg/L)	Resistant	MIC_{50} (mg/L)	MIC_{90} (mg/L)	Range (mg/L)
Amoxicillin	100.0	>256	>256	32- >256	100.0	>256	>256	128- >256	100.0	>256	>256	32- >256	100.0	>256	>256	128- >256	100.0	>256	>256	128- >256	100.0	>256	>256	128- >256
Amoxicillin-Clavulanic acid	85.2	128	>256	1- >256	71.1	32	>256	1- >256	96.7	>256	>256	4- >256	100.0	>256	>256	64- >256	95.7	128	>256	8- >256	91.3	>256	>256	4- >256
Piperacillin	98.6	>256	>256	2- >256	99.3	>256	>256	16- >256	100.0	>256	>256	32- >256	92.3	>256	>256	2- >256	100.0	256	>256	64- >256	91.3	>256	>256	8- >256
Piperacillin-Tazobactam	60.6	128	>256	≤0.125- >256	36.8	4	>256	≤0.125- >256	83.5	>256	>256	2- >256	80.8	256	>256	2- >256	60.9	64	>256	1- >256	69.6	128	>256	≤0.125- >256
Cefoxitin[a]	48.4	8	>256	1- >256	27.0	4	32	1- >256	51.2	16	128	1- >256	100.0	256	>256	16- >256	95.7	256	>256	4- >256	69.6	64	>256	2- >256
Cefotaxime	96.8	256	>256	≤0.125- >256	96.1	256	>256	≤0.125- >256	98.3	>256	>256	0.5- >256	96.2	128	>256	0.5- >256	100.0	64	256	4- >256	91.3	64	>256	1- >256
Ceftazidime	77.7	64	>256	≤0.125- >256	65.1	16	128	≤0.125- >256	93.4	128	>256	≤0.125- >256	69.2	64	>256	1- >256	87.0	128	>256	2- >256	82.6	64	>256	0.5- >256
Cefepime	66.7	128	>256	≤0.125- >256	66.4	32	>256	≤0.125- >256	89.3	>256	>256	≤0.125- >256	30.8	2	256	≤0.125- >256	13.0	2	16	≤0.125- >256	43.5	4	>256	≤0.125- >256
Aztreonam	82.3	64	>256	≤0.125- >256	81.6	32	256	≤0.125- >256	88.4	256	>256	≤0.125- >256	65.4	32	128	≤0.125- >256	91.3	32	256	≤0.125- >256	65.2	16	>256	≤0.125- >256
Imipenem	7.2	0.25	4	≤0.125- 256	3.9	≤0.125	0.5	≤0.125- 128	4.1	0.5	4	≤0.125- 256	7.7	0.25	4	≤0.125- 16	0.0	0.5	1	0.25- 1	13.0	1	8	≤0.125- 16
Meropenem	4.1	≤0.125	2	≤0.125- 256	0.7	≤0.125	≤0.125	≤0.125- 32	8.3	0.25	4	≤0.125- 256	3.8	≤0.125	4	≤0.125- 16	0.0	≤0.125	≤0.125	≤0.125- 0.25	4.3	≤0.125	8	≤0.125- 16
Ertapenem	25.8	≤0.125	8	≤0.125- >256	5.9	≤0.125	0.25	≤0.125- 16	52.9	2	16	≤0.125- >256	38.5	0.5	16	≤0.125- 128	8.7	0.25	0.5	≤0.125- 4	17.4	≤0.125	2	≤0.125- 8
Gentamicin	38.3	1	128	≤0.125- >256	25.0	0.5	64	≤0.125- >256	62.0	32	128	≤0.125 ->256	11.5	0.25	8	≤0.125- 128	26.1	0.5	64	≤0.125- 128	47.8	4	128	≤0.125- 256
Tobramycin	42.6	2	32	≤0.125- >256	27.6	0.5	32	≤0.125- >256	69.4	16	32	≤0.125- >256	11.5	0.5	16	≤0.125- 64	34.8	0.5	16	≤0.125- 64	47.8	4	32	≤0.125- 32
Amikacin	2.9	2	8	≤0.125- >256	2.6	2	4	≤0.125- >256	4.1	2	8	≤0.125- >256	0.0	1	2	≤0.125- 16	0.0	1	2	0.5- 16	0.0	1	8	0.25- 16
Netilmicin	37.7	1	32	≤0.125- >256	27.0	0.5	16	≤0.125- >256	58.7	8	16	≤0.125- >256	11.5	0.25	16	≤0.125- 32	26.1	0.25	32	≤0.125- 128	43.5	4	32	≤0.125- 256
Arbekacin	NA	0.5	2	≤0.125- >256	NA	0.5	2	≤0.125- >256	NA	1	4	≤0.125- >256	NA	0.25	1	≤0.125- >256	NA	0.25	2	≤0.125- 8	NA	1	4	≤0.125- 16
Nalidixic acid	NA	>256	>256	1- >256	NA	>256	>256	1- >256	NA	>256	>256	2- >256	NA	4	>256	2- >256	NA	16	>256	2- >256	NA	128	>256	2- >256
Ciprofloxacin	66.7	16	>256	≤0.125- >256	66.4	16	>256	≤0.125- >256	85.1	64	>256	≤0.125- >256	19.2	≤0.125	16	≤0.125- 64	43.5	0.5	32	≤0.125- 64	73.9	16	>256	≤0.125- >256
Levofloxacin	63.2	8	64	≤0.125- >256	88.2	16	>256	0.25- >256	76.9	8	64	≤0.125- >256	19.2	8	4	≤0.125- >256	43.5	0.5	32	≤0.125- 64	52.2	8	>256	≤0.125- >256
Trimethoprim-Sulfamethoxazole	71.9	>256	>256	≤0.125- >256	71.7	>256	>256	≤0.125- >256	84.3	>256	>256	≤0.125- >256	38.5	0.5	>256	≤0.125- >256	52.2	128	>256	≤0.125- >256	69.6	256	>256	≤0.125- >256
Tigecycline	54.8	1	16	≤0.125- >256	38.2	0.5	8	≤0.125- >256	73.6	2	16	≤0.125- >256	69.2	1	16	≤0.125- >256	13.0	0.5	8	≤0.125- 32	60.9	2	16	≤0.125- 64
Fosfomycin	38.6	16	>256	≤0.125- >256	9.2	4	32	≤0.125- >256	70.2	128	>256	≤0.125- >256	53.8	64	128	≤0.125- >256	26.1	4	>256	0.5- >256	65.2	64	>256	2- >256
Colistin	7.5	≤0.125	1	≤0.125- >256	0.7	≤0.125	0.5	≤0.125- 8	10.7	≤0.125	2	≤0.125- >256	15.4	≤0.125	256	≤0.125- >256	0.0	≤0.125	0.25	≤0.125- 2	34.8	≤0.125	>256	≤0.125- >256

[a] For cefoxitin, the EUCAST ECOFF (>8 mg/L) was used.

NA. Not available (breakpoints have not been established by EUCAST)

Supplementary Table 2. Primers used in the detection of antimicrobial resistance genes and expected amplicon sizes.

**Resistance**	**Gene**	**DNA sequence (5´–3´)**	**Amplicon size (bp)**	**Annealing temperature (ºC)**	**Reference**
β-lactams	bla_{TEM}	ATGAGTATTCAACATTTCCG	867	55	[1]
		CTGACAGTTACCAATGCTTA			
	bla_{SHV}	GGGTTATTCTTATTTGTCGC	930	55	[1]
		TTAGCGTTGCCAGTGCTC			
Cephalosporins	bla_{CTXM}	ATGTGCAGYACCAGTAARGT	593	55	[2]
		TGGGTRAARTARGTSACCAGA			
	$bla_{CTXM\text{-}group1}$	ATGGTTAAAAAATCACTGCG	912	55	[3]
		TTACAAACCGTCGGTGAC			
	$bla_{CTXM\text{-}group9}$	ATGGTGACAAAGAGAGTGCAAC	876	55	[3]
		TTACAGCCCTTCGGCGATG			
	$bla_{CTXM\text{-}group8}$	TCGCGTTAAGCGGATGATGC	666	55	[4]
		AACCCACGATGTGGGTAGC			
Cephamycinases	bla_{ACC}	AACAGCCTCAGCAGCCGGTTA	346	64	[5]
		TTCGCCGCAATCATCCCTAGC			
	bla_{CIT}	TGGCCAGAACTGACAGGCAAA	462		[5]
		TTTCTCCTGAACGTGGCTGGC			
	bla_{DHA}	AACTTTCACAGGTGTGCTGGGT	405		[5]
		CCGTACGCATACTGGCTTTGC			
	bla_{MOX}	GCTGCTCAAGGAGCACAGGAT	520		[5]
		CACATTGACATAGGTGTGGTGC			
	bla_{EBC}	TCGGTAAAGCCGATGTTGCGG	302		[5]
		CTTCCACTGCGGCTGCCAGTT			
	bla_{FOX}	AACATGGGGTATCAGGGAGATG	190		[5]
		CAAAGCGCGTAACCGGATTGG			
Carbapenems	$bla_{OXA\text{-}48}$	TGCGTGTATTAGCCTTATCG	784	60	[6]
		TTTTTCCTGTTTGAGCACTTC			
	bla_{IMP}	GAAGGCGTTTATGTTCATAC	586	55	[7]
		GTAAGTTTCAAGAGTGATGC			
	bla_{KPC}	CATTCAAGGGCTTTCTTGCTGC	538	60	[8]
		ACGACGGCATAGTCATTTGC			
	bla_{NDM}	CCATGCGGGCCGTATGAGTGATTG	700	60	[9]
		TCGCGAAGCTGAGCACCGCATTAG			
	bla_{VIM}	ATGGTGTTTGGTCGCATATC	510	60	[10]
		TGGGCCATTCAGCCAGATC			

References

[1] Rasheed JK, Jay C, Metchock B, Berkowitz F, Weigel L, Crellin J, Steward C, Hill B, Medeiros AA, Tenover FC. Evolution of extended-spectrum beta-lactam resistance (SHV-8) in a strain of *Escherichia coli* during multiple episodes of bacteremia. Antimicrob Agents Chemother. 1997 Mar;41(3):647-53.

[2] Pagani L, Dell'Amico E, Migliavacca R, D'Andrea MM, Giacobone E, Amicosante G, Romero E, Rossolini GM. Multiple CTX-M-type extended-spectrum beta-lactamases in nosocomial isolates of *Enterobacteriaceae* from a hospital in northern Italy. J Clin Microbiol. 2003 Sep;41(9):4264-9.

[3] Ruiz del Castillo B, Vinué L, Román EJ, Guerra B, Carattoli A, Torres C, Martínez-Martínez L. Molecular characterization of multiresistant *Escherichia coli* producing or not extended-spectrum β-lactamases. BMC Microbiol. 2013 Apr 16;13:84

[4] Woodford N, Fagan EJ, Ellington MJ. Multiplex PCR for rapid detection of genes encoding CTX-M extended-spectrum (beta)-lactamases. J Antimicrob Chemother. 2006 Jan;57(1):154-5.

[5] Perez-Perez, F.J. Hanson, N.D. Detection of plasmid-mediated AmpC beta-lactamase genes in clinical isolates by using multiplex PCR. Journal of clinical microbiology (2002) 40, 2153-2162.

[6] Oteo J, Saez D, Bautista V, Fernández-Romero S, Hernández-Molina JM, Pérez-Vázquez M, Aracil B, Campos J; Spanish Collaborating Group for the Antibiotic Resistance Surveillance Program. Carbapenemase-producing *Enterobacteriaceae* in Spain in 2012. Antimicrob Agents Chemother. 2013 Dec;57(12):6344-7.

[7] Miró E, Agüero J, Larrosa MN, Fernández A, Conejo MC, Bou G, González-López JJ, Lara N, Martínez-Martínez L, Oliver A, Aracil B, Oteo J, Pascual A, Rodríguez-Baño J, Zamorano L, Navarro F. Prevalence and molecular epidemiology of acquired AmpC β-lactamases and carbapenemases in *Enterobacteriaceae* isolates from 35 hospitals in Spain. Eur J Clin Microbiol Infect Dis. 2013 Feb;32(2):253-9.

[8] Dallenne C, Da Costa A, Decré D, Favier C, Arlet G. Development of a set of multiplex PCR assays for the detection of genes encoding important beta-lactamases in *Enterobacteriaceae*. J Antimicrob Chemother. 2010 Mar;65(3):490-5.

[9] Oteo J, Domingo-García D, Fernández-Romero S, Saez D, Guiu A, Cuevas O, Lopez-Brea M, Campos J. Abdominal abscess due to NDM-1-producing *Klebsiella pneumoniae* in Spain. J Med Microbiol. 2012 Jun;61(Pt 6):864-7.

[10] Poirel L, Naas T, Nicolas D, Collet L, Bellais S, Cavallo JD, Nordmann P. Characterization of VIM-2, a carbapenem-hydrolyzing metallo-beta-lactamase and its plasmid- and integron-borne gene from a *Pseudomonas aeruginosa* clinical isolate in France. Antimicrob Agents Chemother. 2000 Apr;44(4):891-7.
